# Sarcopenic obesity is associated with telomere shortening: findings from the NHANES 1999–2002

**DOI:** 10.1038/s41366-021-00995-z

**Published:** 2021-11-04

**Authors:** Thomas Goddard, Kostas Tsintzas, Blossom C. M. Stephan, Carla M. Prado, Mohsen Mazidi, Mario Siervo

**Affiliations:** 1 School of Life Sciences, Division of Physiology, Pharmacology and Neuroscience, University of Nottingham, Queen’s Medical Centre, Nottingham, UK; 2 Institute of Mental Health, The University of Nottingham Medical School, Nottingham, UK; 3 Human Nutrition Research Unit, Department of Agricultural, Food and Nutritional Science, University of Alberta, Edmonton, Alberta Canada; 4 Department of Twin Research and Genetic Epidemiology, Kings College London, London, UK

**Keywords:** Ageing, Risk factors

## Abstract

Sarcopenic obesity (SO) is characterised by the concurrent presence of sarcopenia and excess adiposity. Telomere shortening has been associated with sarcopenia and obesity alone but the association between SO and telomere length (TL) has not been investigated. This study aimed to investigate SO and TL in an adult population. Data were from 5397 individuals (mean age = 44.7 years, 51.3% male) enrolled in the National Health and Nutrition Examination Survey. Body composition (BC) was assessed by Dual Energy X-Ray Absorptiometry. Two models were used to assess SO: a BC model including four phenotypes derived from the combination of high or low adiposity and muscle mass; and, a truncal fat mass to appendicular skeletal mass ratio (TrFM/ASM). TL was assessed using quantitative polymerase chain reaction and expressed as base pairs. The mean TL, relative to the reference DNA, was calculated and expressed as the mean T/S ratio. A General Linear Model was applied to determine associations between TL for SO. In adjusted analysis, only individuals with SO, defined as the presence of high adiposity-low muscle mass (four-phenotype model), had significantly shorter telomeres (*p* = 0.05) than the reference group (i.e. low adiposity-high muscle mass), with a mean T/S ratio of 1.02 (95%CI: 0.98–1.05) compared to 1.05 (95%CI: 1.01–1.09), respectively. TrFM/ASM was not associated with TL. Preliminary findings suggest that sarcopenia and obesity may act synergistically to shorten telomeres.

## Introduction

Sarcopenic obesity (SO) is defined by the concurrent presence of low muscle mass (sarcopenia) and high adiposity (obesity) [1]. SO appears to confer a greater risk for cardio-metabolic diseases and mortality, than either sarcopenia or obesity alone [1].

Telomeres are DNA-protein structures located at the ends of chromosomes, which protect the genome from inter-chromosomal fusion, nucleolytic degradation, and genome instability [2]. In the absence of sufficient telomerase activity, telomeres are shortened, and this has been used as a biomarker of biological ageing [2]. Individuals with shorter telomeres may have a greater risk of cardio-metabolic disorders and mortality [2]. However, even though telomere shortening has been associated with sarcopenia and obesity separately [3, 4], the link between SO and telomere length (TL) has not been investigated to determine whether SO may represent a greater risk factor for accelerated ageing and age-related cardio-metabolic disorders.

Therefore, the aim of this study was to explore the relationship between SO and TL in an adult population-representative cohort recruited as part of National Health and Nutrition Examination Survey (NHANES).

## Material and methods

### National Health and Nutrition Examination Survey

The data for this study were obtained from the NHANES surveys conducted between 1999 and 2002. Survey procedures are available online at http://www.cdc.gov/nchs/nhanes.htm (accessed March 2021). The survey used a complex, multistage probability sampling design to ensure that the sample was representative of non-institutionalised adult (≥18 years) civilians in the United States (US) [5]. The sample included 7110 individuals. Individuals that had missing data for mean telomere (T/S) ratio (*n* = 1712) or had a coefficient of variation for mean T/S ratio (mean T/S ratio/standard deviation of mean T/S ratio) that was >1 (*n* = 1), were excluded. The final sample included 5397 individuals.

### Dual energy X-ray absorptiometry (DXA)

Body composition (BC) was assessed by DXA using a Hologic QDR-4500A. Participants were excluded if they were pregnant or they did not fit on the DXA scanner (weight > 136.1 kg and height > 195.6 cm). Participants were also excluded if they had been exposed to nuclear medicine in the previous three days or radiographic contrast material in the previous seven days.

### Body composition phenotypes

Body composition phenotypes were defined using three separate models: (1) a four-phenotype model; (2) the ratio between truncal fat mass (TrFM) and appendicular skeletal muscle mass (ASM); and (3) body mass index (BMI). The four-phenotype model divides individuals into four categories based on levels of adiposity and muscle mass, and previously established reference curves [6]. The four groups were: low adiposity and high muscle mass (LA-HM); low adiposity and low muscle mass (LA-LM); high adiposity and high muscle mass (HA-HM); and, high adiposity and low muscle mass (HA-LM). Individuals with a HA-LM phenotype were considered as having SO. The TrFM/ASM ratio model divided individuals into three categories based on the centile of TrFM/ASM ratio derived from population-based reference curves [7]. These categories were: <15th centile; 15–84th centile; and, ≥85th centile. SO was defined as a TrFM/ASM ratio ≥85th centile of the reference curve. BMI was calculated by weight (kg)/height^2^ (m). Participants were classified as normal weight (BMI = 18.5–24.9 kg/m^2^), overweight (BMI = 25.0–29.9 kg/m^2^) or obesity (BMI ≥ 30.0 kg/m^2^).

### Telomere length

Telomere length was analysed using whole blood and qPCR and reported as base pairs. The mean TL, relative to the reference DNA, was calculated and expressed as the mean T/S ratio. Assay runs with control values beyond two standard deviations of the mean of all runs, was excluded (<6% of runs). Outliers within samples were identified and excluded (<2% of samples). The interassay coefficient of variation was 6.5%.

### Statistical analysis

Statistical analysis was conducted using the SPSS Complex Samples module according to NHANES protocols found online at https://wwwn.cdc.gov/nchs/nhanes/tutorials/Module4.aspx (accessed March 2021). Analysis was prepared by adjusting for strata, clusters, and a 4-year sample weight. Descriptive statistics were calculated through complex analysis unless otherwise stated, and presented as mean with standard error (continuous variables) or as percentages of total sample weight (categorical variables). A General Linear Model (GLM) was applied to the three models of BC, with the mean T/S ratio selected as the outcome variable on each occasion. Analyses were adjusted for age, sex, ethnicity, education, physical activity, smoking, alcohol intake and general health condition, which are covariates that have been described previously [5]. The GLM analysis produced mean T/S values for each phenotype, in addition to 95% confidence intervals (CI), and was used to test for statistical significance between each phenotype and the reference phenotypes (BC = LA-HM; TrFM/ASM ≤ 15th centile; BMI = normal weight). The mean T/S ratio value and corresponding CIs of each phenotype were converted to TL in base pairs (bp) using a formula specific to the assay (3274 + 2413*(T/S)), to support the interpretation of the results. Analyses were conducted using IBM SPSS Version 27 for Windows. A *p* value ≤ 0.05 was considered as statistically significant.

## Results

The study sample included 5397 individuals, with a mean age of 44.7 years (51.3% male). The four-component BC phenotype model classified 25.0% of individuals as LA-HM, 24.7% as LA-LM, 27.9% as HA-HM and 22.4% as HA-LM (SO). The TrFM/ASM model classified 14.7% of individuals as <15th centile, 70.5% as 15–84th centile and 14.7% as ≥ 85^th^ centile (SO). The BMI model classified 33.8% of individuals as normal weight, 36.0% as overweight, and 29.6% as individuals with obesity (Table 1).

**Table 1:** Characteristics of the sample population. Values are through complex analysis unless otherwise stated.

Metric	Value
Participants, *n*^a^	5397
Male, %	51.3
Age, mean (SE)	44.7 (0.4)
BMI (kg/m^2^)	27.7 (0.06)
Ethnicity	
Mexican Hispanic, %	7.3
Other Hispanic, %	7.0
Non-Hispanic White, %	72.7
Non-Hispanic Black, %	9.0
Other Race–Including Multi-Racial, %	3.9
Education (highest level obtained)	
Less Than 9th Grade, %	6.2
9–11th Grade (Includes 12th grade with no diploma), %	13.7
High School Grad/GED or Equivalent, %	26.4
Some College or AA degree, %	28.8
College Graduate or above, %	24.8
Refused, %	<0.1
Don’t Know, %	0.1
Physical activity	
Sits during the day and does not walk a lot, %	23.1
Stands or walks a lot during the day but does not carry or lift things often, %	50.0
Lifts light loads or climbs stairs/hills often, %	18.6
Performs heavy work or carries heavy loads, %	8.2
Refused, %	<0.1
Don’t Know, %	0.1
Alcohol intake, mean gm/day (SE)	12.4 (0.8)
General Health Condition	
Excellent, %	24.2
Very Good, %	33.6
Good, %	28.3
Fair, %	11.5
Poor, %	2.3
Refused, %	0.0
Don’t Know, %	<0.1
Smoking (frequency of cigarette use)	
Every Day, %	42.4
Some Days, %	8.0
Not At All, %	49.6
Refused, %	0.0
Don’t Know, %	0.0
Four-phenotype model classification^b^	
LA-HM, %	25.0
LA-LM, %	24.7
HA-HM, %	27.9
HA-LM (SO), %	22.4
TrFM/ASM ratio classification^c^	
<15th centile, %	14.7
15–84th centile, %	70.5
≥85th centile (SO), %	14.8
BMI classification^d^	
Healthy, %	33.8
Overweight, %	36.0
Obesity, %	29.6
T/S ratio, mean (SE)^e^	1.06 (0.01)

^a^Complex analysis not taken into account.

^b^LA is low adiposity, HA is high adiposity, LM is low muscle mass and HM is high muscle mass.

^c^TrFM is truncal fat mass, ASM is appendicular skeletal mass.

^d^BMI is body mass index.

^e^The mean telomere length, relative to the reference DNA, was calculated and expressed as the mean T/S ratio.

In adjusted analyses, only subjects with the HA-LM phenotype had significantly shorter telomeres than the reference group LA-HM, with a mean T/S value of 1.02 (95%CI: 0.98–1.05, *p* = 0.05) compared to 1.05 (95%CI: 1.01–1.09), respectively. The mean T/S ratio for the HA-LM group corresponded to a TL of 5735 bp (95%CI: 5650–5820), which was significantly shorter (difference: −87 bp, 95%CI: −20 to −145 bp, *p* = 0.05) compared the reference group LA-HM. No significant association was found between TrFM/ASM phenotypes and TL, or between BMI groups and TL (Table 2).

**Table 2:** Mean T/S ratio and telomere length in base pairs for body composition phenotypes, based on four-phenotype and TrFM/ASM model classifications.

	Unadjusted					Adjusted				
	Mean T/S ratio^a^		Telomere length (base pairs)			Mean T/S ratio^a^		Telomere length (base pairs)		
	Mean	95% CI	Mean	95% CI	*P* value	Mean	95% CI	Mean	95% CI	*P* value
BMI										
Normal weight (reference)	1.10	1.07–1.13	5930	5851–6009		1.06	1.03–1.09	5829	5747–5911	
Overweight	1.05	1.02–1.09	5813	5729–5897	**0.003**	1.04	1.00–1.08	5790	5698–5883	0.22
Obesity	1.04	1.01–1.07	5780	5701–5859	**<0.001**	1.04	1.01–1.07	5777	5705–5850	0.07
Four-phenotype model classification^b^										
LA-HM (reference)	1.08	1.05–1.12	5902	5815–5990		1.05	1.01–1.09	5822	5724–5920	
LA-LM	1.04	1.01–1.07	5799	5728–5871	**0.01**	1.04	1.01–1.07	5798	5726–5870	0.61
HA-HM	1.07	1.04–1.11	5870	5784–5957	0.32	1.06	1.02–1.10	5842	5738–5946	0.64
HA-LM (SO)	1.04	1.00–1.07	5790	5708–5872	**0.004**	1.02	0.98–1.05	5735	5650–5820	**0.05**
TrFM/ASM classification^c^										
<15th centile (reference)	1.10	1.05–1.15	5937	5819–6054		1.07	1.02–1.12	5858	5736–5979	
15–84th centile	1.05	1.02–1.08	5829	5754–5903	**0.01**	1.04	1.01–1.07	5797	5721–5874	0.15
≥85th centile (SO)	1.05	1.02–1.08	5818	5750–5885	**0.02**	1.03	0.99–1.07	5767	5678–5857	0.16

Significant results are highlighted in bold.

^a^The mean telomere length, relative to the reference DNA, was calculated and expressed as the mean T/S ratio.

^b^LA is low adiposity, HA is high adiposity, LM is low muscle mass and HM is high muscle mass.

^c^TrFM is truncal fat mass, ASM is appendicular skeletal mass.

## Discussion

These preliminary results suggest that SO may be associated with telomere shortening and may represent an important risk factor for accelerated ageing than sarcopenia and obesity alone. Sarcopenia and obesity have been associated individually with greater inflammation and generation of reactive oxygen species [2, 8], which are linked to single-strand breaks in DNA and have a high affinity for the G-rich fragments within telomeres [9]. Hence, the co-occurrence of sarcopenia and obesity may exacerbate these processes and lead to greater telomere shortening.

The TrFM/ASM model found no significant association between SO and TL; this result was unexpected as truncal fat accumulation has been associated with greater impairment of cardio-metabolic health [10]. Further research is needed to investigate whether whole-body fat mass and truncal fat mass may differ in their associations with mechanisms related to telomere shortening and ageing processes.

The stratification of the population by the four-component BC phenotype model showed a significant association in adjusted models between the SO (HA-LM) phenotype and TL whereas no significant association was found between BMI groups and telomere shortening. These findings are contrasting with previous literature indicating a significant association between BMI and telomere shortening; a potential reason for the difference could be attributed to the difference in sample size as the previous analysis was based on eighty-seven distinct study samples were including 146,114 individuals [4]. The key observation of this study is that the concurrence of sarcopenia and obesity may produce a greater, synergistic effect on telomeres than either sarcopenia or obesity alone. Mechanisms that could explain telomere shortening include increased oxidative stress, endocrine dysfunction and chronic inflammation [11] and previous studies have reported greater levels of oxidative stress and inflammation in the SO phenotype compared to sarcopenia and obesity alone [12, 13].

This study has numerous strengths. This analysis included a large number of individuals that were multi-ethnic and representative of the adult civilian population in the US. BC was assessed using DXA, which is currently the preferred field method of assessment based upon accuracy and reliability. The analyses were also adjusted for demographic, health and lifestyle covariates. TL was measured by a well-established protocol, which included stringent quality control measures such as the identification and exclusion of outlying values. However, this study also has limitations. Subjects were excluded from DXA-assessment if they weighed over 136.1 kg or were taller than 195.6 cm, which may render these findings unrepresentative of those with extreme body types. The classification of sarcopenia did not include the assessment of muscle function, which is a distinct component of sarcopenia assessment. It was not possible to investigate a causal association between BC phenotypes and telomere shortening due to the cross-sectional design of the study.

In conclusion, these preliminary results indicate a significant association between SO and telomere shortening and may suggest that sarcopenia and obesity may act synergistically on the ageing process. Future work should be conducted to further explore these associations in longitudinal cohorts to understand whether SO may be associated with a greater rate of telomere shortening.
